# Learning From Each Other in the Management of Natural Disaster and COVID-19 Pandemic: A Case Study in Taiwan

**DOI:** 10.3389/fpubh.2021.777255

**Published:** 2021-12-09

**Authors:** Hsiao-Wen Wang, Guan-Wei Chen, Wei-Lin Lee, Shuei-Huei You, Chia-Wen Li, Jiun-Huei Jang, Chjeng-Lun Shieh

**Affiliations:** ^1^Department of Hydraulic and Ocean Engineering, National Cheng Kung University, Tainan, Taiwan; ^2^Department of Internal Medicine, National Cheng Kung University Hospital, Tainan, Taiwan

**Keywords:** COVID-19, communicable disease, natural disaster, disaster management, emergency operation

## Abstract

In response to the COVID-19 pandemic, Taiwan has been one of the best performers in the world with extremely low infections and deaths. This success can be attributed to the long experiences dealing with natural disasters and communicable diseases. However, with different disastrous characteristics, the disaster management systems for communicable diseases and natural disasters are very different in terms of laws, plans, frameworks, and emergency operations. Taking the response to COVID-19 pandemic as a study subject, we found that disaster management for communicable diseases can be improved through a comparison with natural disasters, and vice versa. First, having wider and longer impacts than natural disasters, the plans and framework for communicable diseases in Taiwan focus more on national and regional scales. Local governments would need more capacity support including budgets and training to conduct investigations and quarantine during the COVID-19 pandemic. Second, for quick response, the emergency operation for communicable diseases was designed to be more flexible than that for natural disasters by giving the commander more authority to adjust to the circumstances. The commanding system requires a more objective consultation group to prevent arbitrary decisions against the COVID-19 pandemic. Finally, risk governance is important for communicable diseases as well as for natural disasters. Additional efforts should be made to enhance vulnerability assessment, disaster reduction, and risk communication for shaping responses and policies in an efficient and coordinating way.

## Introduction

The novel coronavirus disease 2019 (COVID-19) pandemic, caused by the severe acute respiratory syndrome coronavirus 2 (SARS-CoV-2), and named by the International Committee on Taxonomy of Viruses (1), has been rapidly spread worldwide since the end of 2019 and has resulted in great impacts on health, society, economics, and the environment (2–4). To slow down the spread of this epidemic, many countries have implemented non-pharmaceutical intervention policies such as travel restrictions, social distancing policies, quarantine, and lockdowns (5). Nevertheless, by Jun 30, 2021, the infections of COVID-19 continued to rise with over 182 million confirmed cases and 3.9 million deaths in over 190 countries (6).

**Figure 1:**
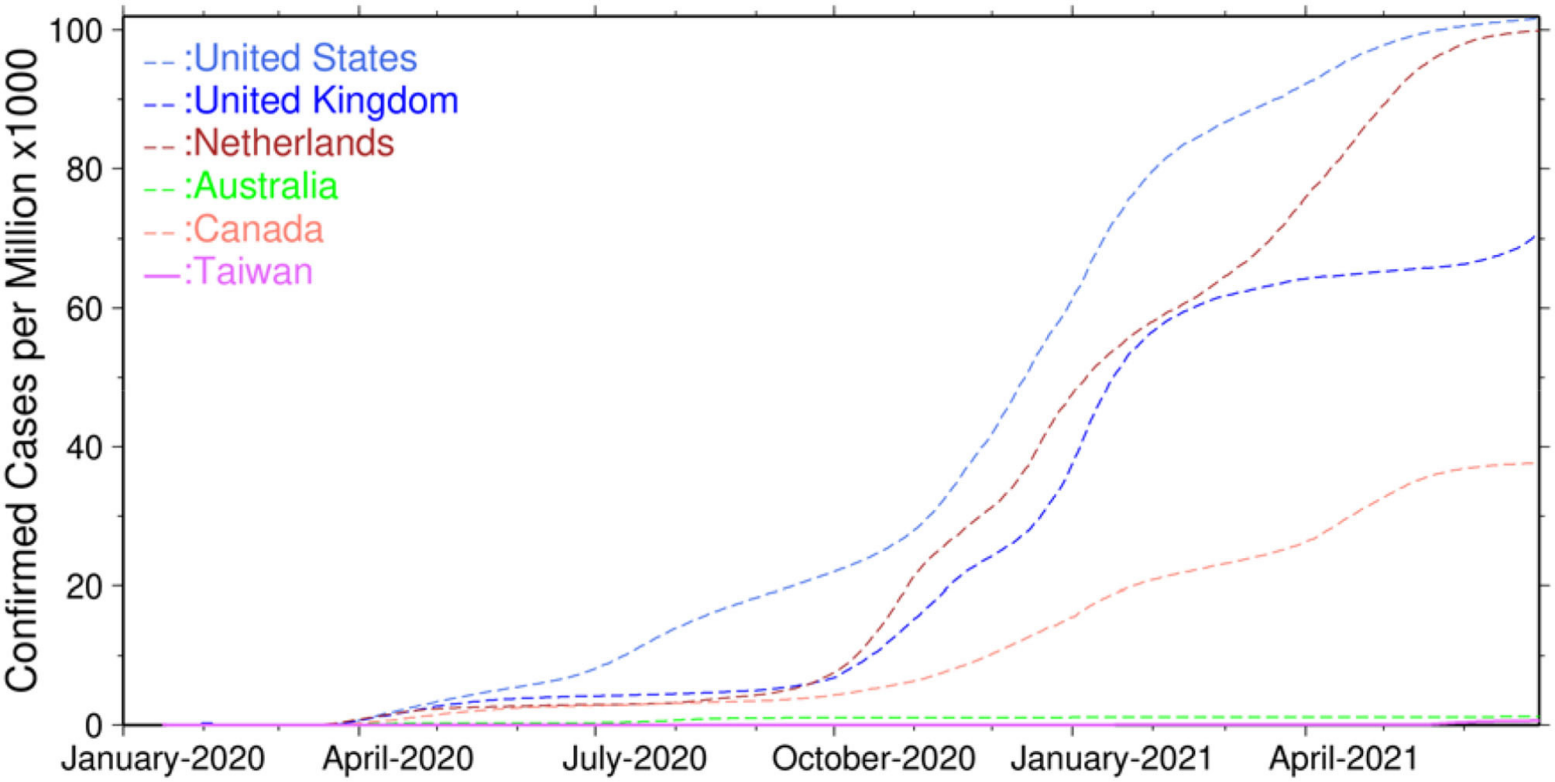
Confirmed infections of COVID-19 worldwide.

Taiwan is one of the best performers in confronting the COVID-19 pandemic in the world. According to the COVID-19 infection statistics in Figure 1, Taiwan had only 1,290 infections and 12 deaths in total (54.16 infections and 0.50 deaths per million) before May 14, 2021. Although Taiwan experienced a sudden rise of infections to 14,804 people (621.58 infections and 27.21 deaths per million) in the period of May 15 to June 30, 2021, the overall infections are still much lower than those in the United States (101,706 infections and 1,827 deaths per million), the United Kingdom (70,962 infections and 1,891 deaths per million), the Netherlands (99,957 infections and 1,052 deaths per million), Australia (1,202 infections and 36 deaths per million), and Canada (37,699 infections and 696 deaths per million).

Taiwan was one of the first to respond to the COVID-19 pandemic by inspecting passengers for fever or pneumonia symptoms on direct flights from Wuhan since COVID-19 was firstly reported in December 2019 (7, 8). On January 5, 2020, Taiwan Centers for Disease Control (TCDC) informed the passengers who had traveled to Wuhan within the previous 14 days with symptoms of an upper respiratory tract infection (URTI) to conduct quarantine at home or in a hospital if medical attention was necessary. On January 20, 2020, a level-3 epidemic command center was activated and implemented relevant strategies such as laboratory diagnosis, border control, community transmission control, medical system response and preparedness, stockpile and allocation of personal protection equipment, health education, and disinformation management (details can be found from the website of the CDC at https://www.cdc.gov.tw). Some advanced technologies were applied to monitor the COVID-19 pandemic, including a mask-rationing plan to limit personal mask purchase in the beginning of pandemic outbreak, a digital fence system to monitor if someone does not stay in the assigned place during quarantine, a communication APP based on LINE chatbot to answer questions automatically, and a short-message-service (SMS) real-name registration system to track someone's footprint if an infection is confirmed. These strategies and advanced technologies successfully suppressed the infections to a very low level in Taiwan for more than 1 year. However, the COVID-19 infection finally broke through the line after the outbreak of a cluster infection at a quarantine hotel in May 2021 due to the loose isolation and triage measures, lack of vaccination, and insufficient widespread testing.

Disasters involve many aspects of community, government, and nongovernmental functions. In fact, Taiwan has established comprehensive disaster management systems based on decades-long experience of fighting with natural disasters and communicable disasters (9). While communicable disasters are categorized as biological disasters in Taiwan, in the response to the COVID-19 pandemic, we found that the disaster management systems and corresponding operations for communicable disasters are very different from those for natural disasters. The COVID-19 crisis is considered as a long-term public health emergency rather than most natural disasters with temporary damages to the built environment such as flooding. This is because the scales of life loss, economic impact, and social disruption caused by COVID-19 pandemic are much larger than those caused by natural disasters. Taking Taiwan as an example, the international communications of academy, commerce, and travel have stagnated for nearly 2 years since the implementation of border control in March 2020. This was never happened for natural disasters such as floods, typhoons, and landslides which normally lasted for serval days and only caused traffic interruption between local communities. While some impacts caused by COVID-19 stop, the others may continue in different forms. Understanding how the two disaster management systems are structured and functioned is crucial for shaping responses and policies in a more efficient and coordinating way.

The comparison of management systems for natural disasters and COVID-19 diseases has been conducted by a few researchers (10–12). Some studies showed that there are conflicts between the two systems that need to be fixed, whereas some indicated that one system may benefit from the other. For example, Simonovic et al. (13) pointed out that maintaining social distance can be very difficult during emergency evacuation for natural disasters in the COVID-19 pandemic period because the former requires collaboration but the latter requires isolation. Ishiwatari et al. (14) indicated that existing disaster-management measures should be restructured to protect human life and security during the COVID-19 pandemic, which required health and disaster related organizations to coordinate and share information based on scientific knowledge (15). Dzigbede et al. (16) showed that the existence of preparedness, response, and recovery mechanisms for natural disasters helps to combat the COVID-19 pandemic through the investigation of local government capacity in the United States. Among the Caribbean islands, Hambleton et al. (17) indicated that the slowing down of COVID-19 spreading can be attributed to the early border controls issued under the collaborative framework of Caribbean Community (CARICOM), established since 1973 in response to seasonal hurricane threats.

Taking the COVID-19 epidemic experience in Taiwan as an example, this study aims to compare the disaster management systems as well as the experiences in responses and operations for communicable disease and natural disasters in terms of the evolution of laws, plans, frameworks, and emergency operations. Through an overall inspection and mutual learning, lessons can be learned to improve the disaster management systems for both communicable disease and natural disasters. The findings will be valuable for other regions in the response to COVID-19 pandemic during subsequent waves.

## Methods

This study aims to explore the structures and functions of the emergency management systems in Taiwan and how they influence the responses to natural disasters and COVID-19. First, we comprehensively reviewed the corresponding laws, regulations, operational plans, program reports, literatures, and official press releases for communicable diseases and natural disasters. Then, we conducted interviews with experts and officials from central and local governments involved in the disaster management system in Taiwan as well as local village representatives for their inputs on identifying the lessons that should be learned. The selection of interviewees was based on the representativeness of the roles and experiences in dealing with natural disasters or pandemics. The main inclusion criteria is that the participants must have been involved in managing natural disasters or pandemics for at least 3 years. The backgrounds of the interviewees are listed in Table 1. Each interview lasted half-an-hour to 2 h depending on each interviewee's time availability. In total, two from central government, six from local government, and three local villagers were interviewed. Some key interview questions are listed as below:

- Based on your experience, what are the differences between natural disasters and biological disasters in terms of characteristics and disaster responses?
- Have you observed any conflict or inconsistency between Disaster Prevention and Response Act and Communicable Disease Control Act? If yes, what are they?
- How do you follow Disaster Prevention and Response Act and Communicable Disease Control Act in your professional responsibility during the COVID-19 pandemic? Please try to describe in terms of disaster prevention, mitigation, preparedness, response, and recovery.
- Do you know the regulations of Central Epidemic Command Center, and its legalized timing, level, and organization of establishment? How about the Central Emergency Operation Center?
- What do you think about the performance of Central Epidemic Command Center so far?
- What are the interactions have you observed or been involved in between the Office of Disaster Management and the Health Bureaus during the COVID-19 pandemic?
- Do you know All-Hazard Approach in managing disasters? Would you prefer it to the current management system? Why or why not?

**Table 1:** Backgrounds of interviewees.

**Code**	**Position**	**Field**
CG1	Central Government	Natural Disaster
CG2	Central Government	Natural Disaster and COVID-19
LG1	Local Government	Natural Disaster and COVID-19
LG2	Local Government	Natural Disaster and COVID-19
LG3	Local Government	Natural Disaster and COVID-19
LG4	Local Government	COVID-19
LG5	Local Government	Natural Disaster
LG6	Local Government	Natural Disaster
LV1	Local Village	Not Applicable
LV2	Local Village	Not Applicable
LV3	Local Village	Not Applicable

## Interview Results

The suggestions and feedbacks from the interviewees in Table 1 are summarized as below:

- Although Taiwan had experiences dealing with SARS in 2003, the current pandemic provides many lessons to learn (CG 1, CG2, LG1, LG2, LG3, LG4).
- The establishment and operations of emergency operation center and epidemic command center are two entirely different systems (CG 1, CG2, LG1, LG2, LG3, LG4, LG5, LG6).
- Being two different systems, the roles and tasks in dealing with these two disasters are sometimes overlapped or conflicted. It requires intensive communications and coordination. A commander's experiences plays an important role (LG1, LG2, LG3, LG4).
- COVID-19 competes medical capacity of other diseases and reduces the willingness of non-COVID patients to seek medical treatment (LG1, LG2).
- Medical resources can be well allocated across regions under the current system, highlighting the importance of regional network (LG1, LG2, LG3).
- The disaster management framework for communicable diseases does not include the township level as it does for natural disasters. In response to the pandemic, township officials are required to assist investigations and arrange medical treatment which might be beyond their capacity. It appears township officials do not necessarily have sufficient training due to lack of resources (LG3, LG4, LV1, LV2).
- Risk communication is critical. The daily press conference held by Central Epidemic Command Center receives public attention widely, however the released information focused more on numbers of confirmed cases and their travel histories, rather than a holistic risk assessment (CG1, CG2, LG5, LG6).
- Translating knowledge into languages that public can understand is important and still needs more efforts (CG1, CG2, LG1, LG2, LG3, LG4, LG5, LG6, LV1, LV2, LV3).
- Risk maps can be a useful tool to better allocate resources (CG1, CG2, LG1, LG6).
- Taiwan may consider all-hazard approach as a whole, especially for the response stage (CG1, LG1).

## Evolution of the Disaster Management Systems in Taiwan

### Natural Disasters

**Figure 2:**
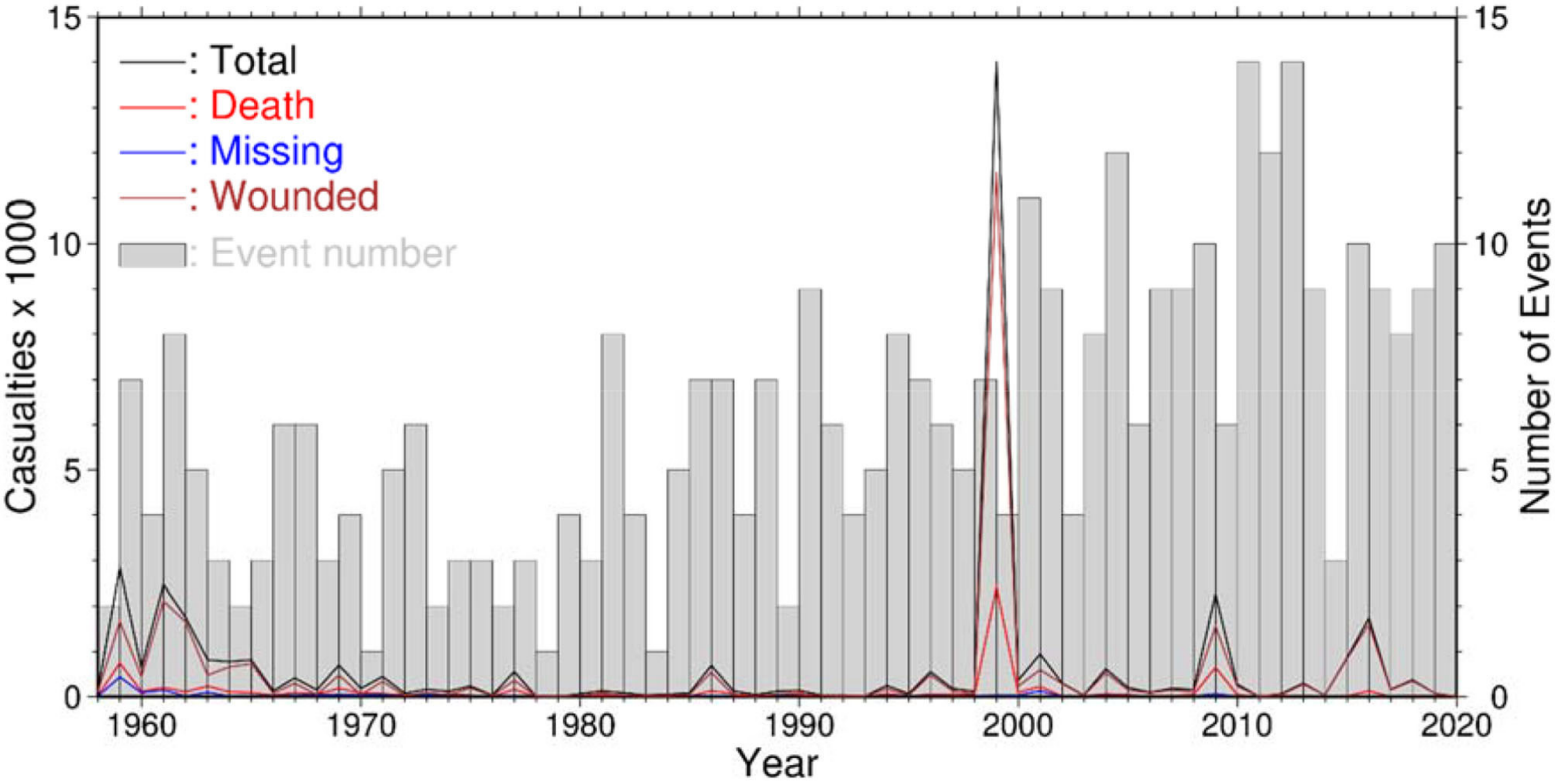
Annual number of natural disasters and corresponding casualties from 1958 to 2019.

**Figure 3:**
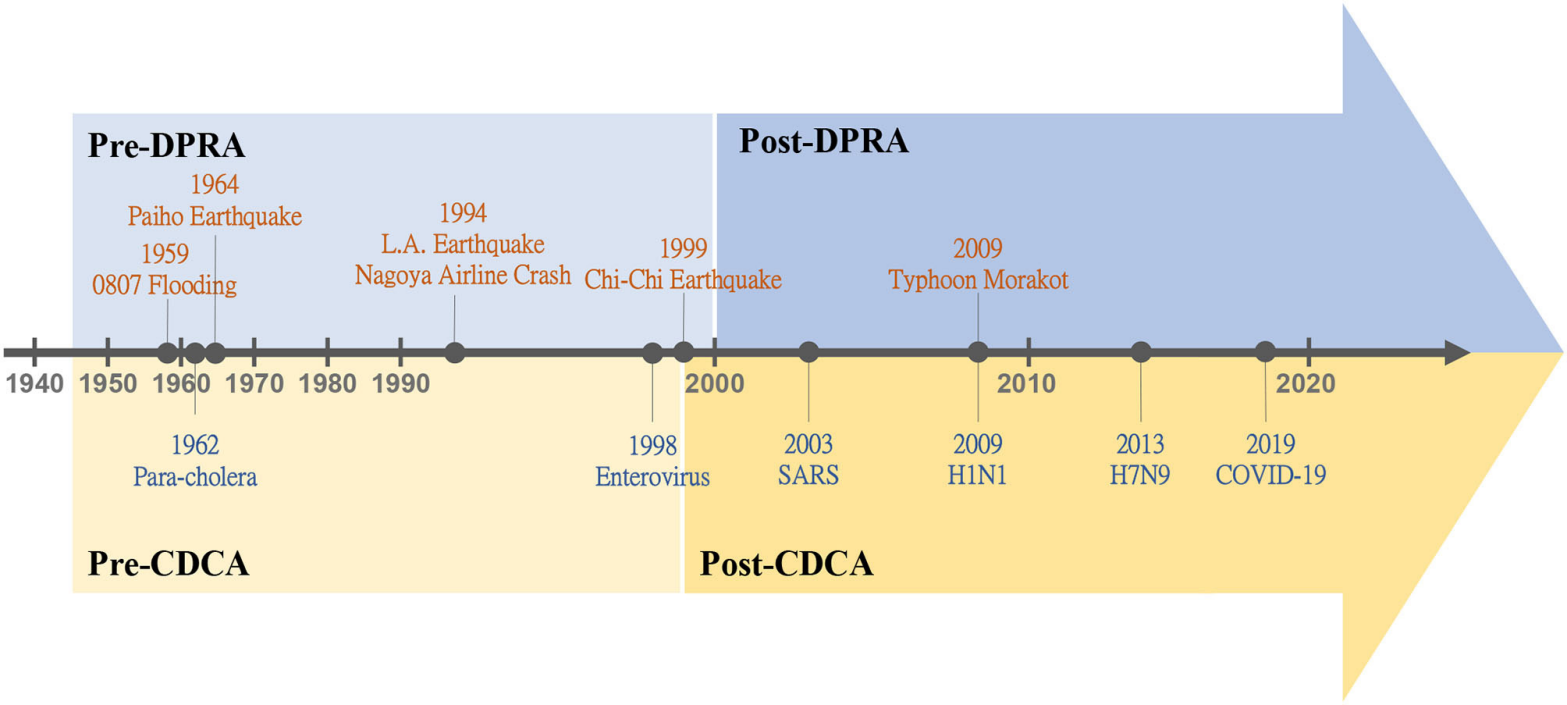
The evolutions of disaster management systems for natural disasters and communicable diseases in Taiwan (where DPRA represents Disaster Prevention and Response Act; CDCA represents Communicable Disease Control Act).

Taiwan is one of the most vulnerable areas in the world suffering from various kinds of natural disasters (18) including typhoons, floods, earthquakes, and landsides which have caused many casualties annually as shown in Figure 2 (19). In the figure, the annual events of natural disaster showed an increasing trend after 2000 but the casualties did not increase simultaneously. This can be attributed to the passing of Disaster Prevention and Response Act (DPRA) in 2000, which enhanced the management system and reduced the impacts caused by natural disasters. Figure 3 shows the evolutions of the disaster management and the disaster history in Taiwan, which can be divided into two periods: the pre-DPRA period from 1945 to 1999 and the post-DPRA period after 2000. During the early stages of the pre-DPRA period, Taiwan experienced several severe natural disasters, two of which were the flooding on August 7, 1959 and the Paiho earthquake on January 18, 1964. The resulted casualties were 1,075 and 756, respectively. The government focused on providing subsidies to the victims affected by natural-disaster-induced casualties and house collapses through executive orders. In 1994, the government realized that the subsidy-based policy may be inadequate in response to devastating disasters such as the Los Angeles earthquake (60 deaths and 9,000 injuries) and the China Airlines' flight crash in Nagoya (264 fatalities). The Executive Yuan proposed the Disaster Prevention and Protection Plan (DPPP) in the same year to deal with prevention, responding, and recovery relating to natural and man-made disasters.

On September 21, 1999, the Chi-Chi earthquake hit central Taiwan, causing 2,415 deaths, 30 missing, 11,305 injuries, 11,000 house collapses, and countless infrastructure damages, with total economic loss amounting up to 12 billion US dollars. This devastating earthquake promoted the legislation of the first disaster management law, the DPRA, in 2000. On August 8, 2009, the heavy rainfall brought by Typhoon Morakot resulted in severe landslides, flooding, and 664 deaths. The catastrophic disaster led to an amendment of the DPRA in which the Office of Disaster Management was established with full-time employees to supervise and implement the policies of national disaster management. More details about the history of natural disaster management in Taiwan can be obtained from Chuang and Ho (9).

### Communicable Disease

In Figure 3, disaster management for communicable disease in Taiwan can be divided into two periods: the pre-CDCA (Communicable Disease Control Act) period from 1945 to 1998 and the post-CDCA period after 1999. In the pre-CDCA period, there were several major communicable disease events in Taiwan, such as the para-cholera outbreak in 1962 and the enterovirus outbreak in 1998. To deal with these epidemics, the Department of Health (DOH) established in 1971 as a directorate general for health affairs was upgraded to the Ministry Of Health and Welfare (MOHW) in 2013. The DOH established several subordinate organizations to implement epidemic prevention works, including the Department of Epidemic Prevention (DEP), the Institute of Preventive Medicine (IPM), and the General Quarantine Office (GQO). In the pre-CDCA period, all policies and measures for epidemic preventions were regulated by the Communicable Disease Control Regulations (CDCR).

In 1999, the CDCR was revised and renamed as the CDCA, and the DEP, GQO, and GQO were combined into the TCDC and became the authority of disease control to take charge of disease prevention, quarantine, surveillance, and inspection in Taiwan. In 2003, the severe acute respiratory syndrome (SARS) struck Taiwan with a total of 668 likely infected cases and 181 deaths (20). The Taiwan government undertook a series of countermeasures that successfully stopped the spread of the epidemic, such as quarantine, community surveillance, and infection prevention network (13, 21). During the next year, the National Health Command Center (NHCC) was established to bridge the information among central, regional, and local authorities in support of decision making during epidemic times. The NHCC has made great contributions during several serious epidemics in recent years, such as the H1N1 Influenza outbreak in 2009, the H7N9 Influenza epidemic in 2013, the Dengue Fever epidemic in 2015, and the current COVID-19 pandemic, as shown in Figure 3.

## Comparisons Between the Disaster Management Systems for Natural Disasters and Communicable Diseases

Based on the evolution of disaster management systems in Taiwan, the comparison between natural disasters and communicable diseases on their laws and plans, management framework, and emergency responses are described as below.

### Laws and Plans

In Taiwan, the disaster management systems for all disasters are regulated by the DPRA, in which each disaster is governed by one ministry when it comes to policy planning and emergency operation. For example, the Ministry of the Interior (MOI) is in charge of typhoon and earthquake disasters, the Ministry of Economic Affairs (MOEA) is responsible for flood disasters, the MOHW takes care of biological disasters, and the Ministry of Transportation and Communications takes care of air crash disasters. The corresponding ministries may propose different plans for different disasters in terms of mitigation, preparedness, response, and recovery. According to the DPRA, the Executive Yuan should announce the basic DPPP as the principal guideline for central ministries to establish disaster-based operational DPPPs and for local governments to follow and establish their own local DPPPs. In other words, the DPRA and basic DPPP provide general rules for all kinds of disasters, whereas the operational and local DPPPs are specific regulations for different disasters and areas. Hence, the budget and penalty for managing different disasters are different. For instance, the fine for spreading fake news for biological disasters is USD 180,000, which is much higher than that for natural disasters as of USD 36,000. According to the operational DPPPs, the annual budget for biological disaster prevention is 22 million USD, whereas that for typhoon disaster prevention is 4.3 million USD.

Based on the DPRA, disaster management in Taiwan includes four phases: mitigation, preparedness, response, and recovery. For each disaster, the responsible ministry should propose related plans to strengthen disaster management in each of the four phases. These laws and regulations work mainly under a command-and-control model as authorities could design a plan and expect people to follow it. For instance, the MOI implemented Disaster Management Capacity Building Plans in 2009 to increase the abilities and capacity of prevention, protection, and collaboration across county, township, and village levels against typhoons (22). To scientifically support the plans proposed by different ministries, the Ministry of Science and Technology has promoted a series of major plans to develop key technologies for disaster prevention and reduction, including the National Science and Technology Program for Hazard Mitigation from 1999 to 2006, the Program on Strengthening the Technology for Disaster Prevention, Reduction, and Implementation from 2007 to 2010, the Program on Applying Science and Technology for Disaster Reduction from 2011 to 2018, and the Innovative Service Program for Disaster Prevention and Reduction Technology from 2019 to 2022. In these programs, updated technologies and platforms are used to bridge the research energy of academic units, the resources of the central government, and the needs of local governments.

In 2016, communicable diseases were officially categorized as one kind of biological disaster and since after regulated by the MOHW under DPRA. Interestingly, according to the operational DPPP for biological disasters, policy plaining and emergency response for communicable diseases should follow the instructions of the CDCA. This makes the CDCA a special act even though biological disasters are regulated by DPRA. In the last 20 years, the MOHW has implemented several national plans for communicable disease control, namely the National Influenza Pandemic Preparedness Plan from 2005 to 2021, the Acute Infectious Disease Risk Monitoring and Management Plan from 2014 to 2019, and the Plan for preparing and response to severe infectious pneumonia during the COVID-19 period.

**Figure 4:**
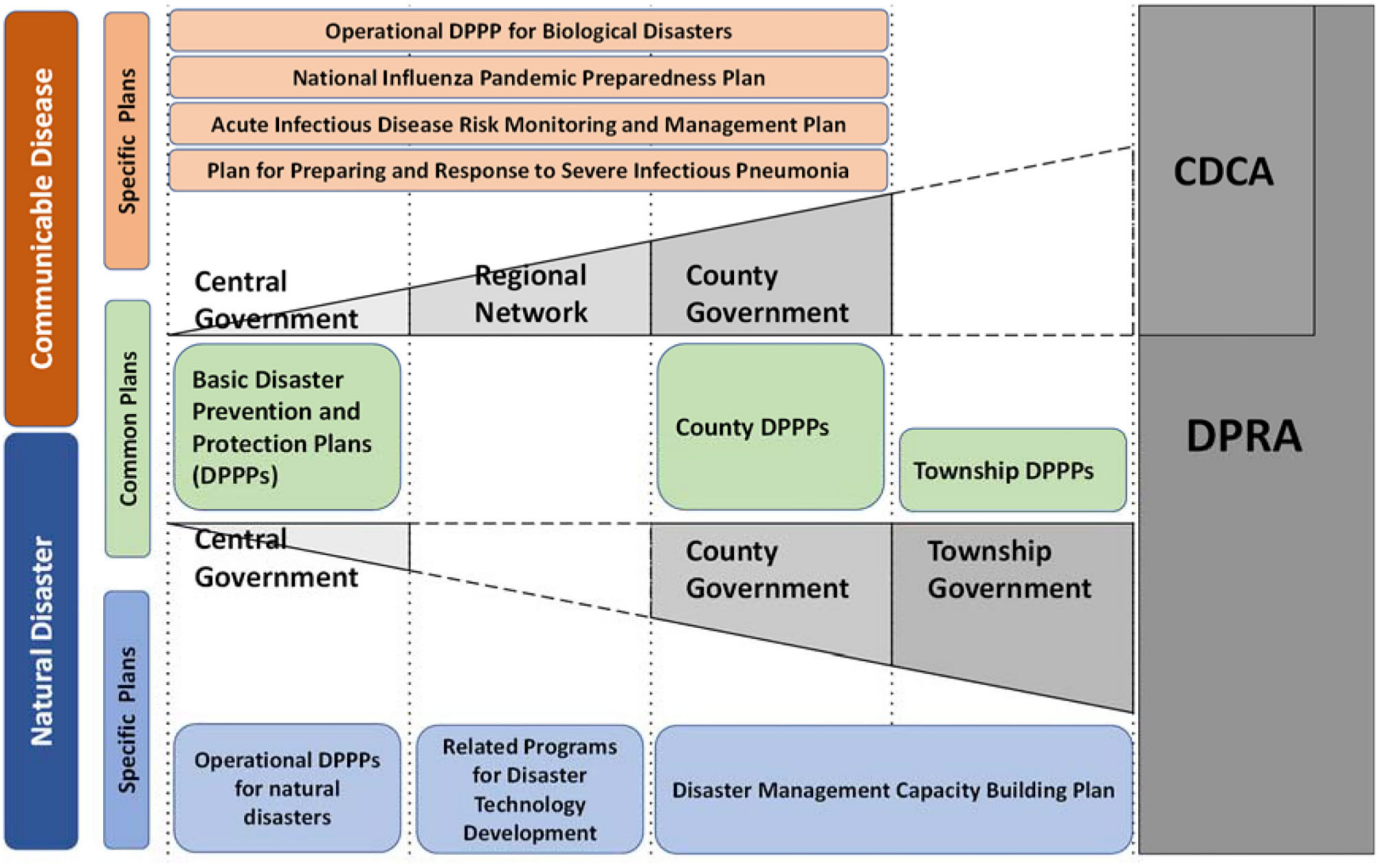
The laws and plans for natural disasters and communicable diseases.

The laws and plans for natural disasters and communicable diseases are summarized in Figure 4. Compared to the plans for natural disasters with an equal emphasis on the four phases, the plans for communicable diseases focus more on epidemic preparedness and response. In addition, the plans for natural disasters root deep into the township and village levels, whereas the plans for communicable diseases emphasize collaboration on national and regional scales. The main reason is that local efforts and awareness are critical in response to immediate disasters such as flooding, while communicable diseases, such as COVID-19, can easily spread across regions and nations which makes regional coordination more important.

### Framework

**Figure 5:**
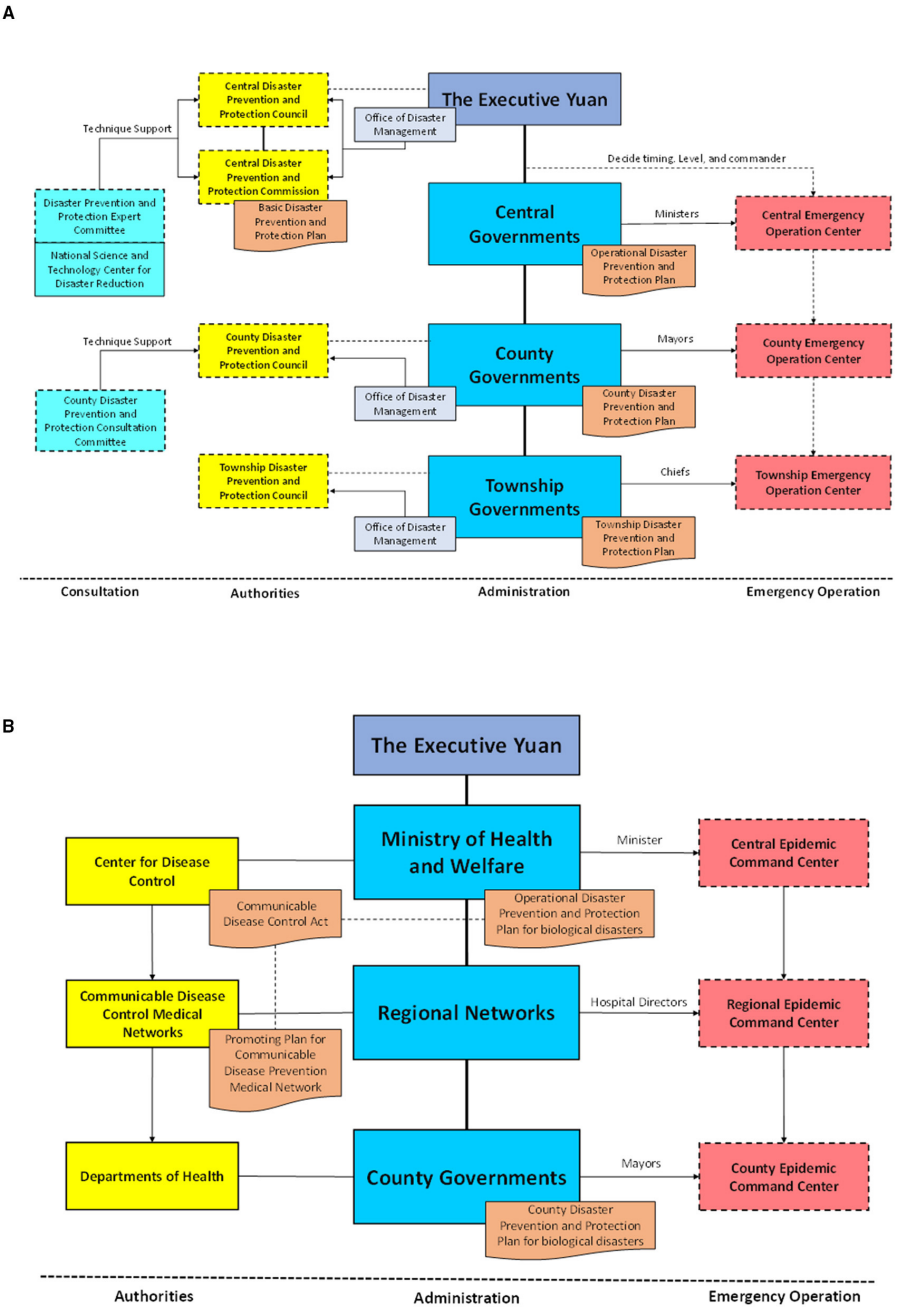
Disaster management frameworks in Taiwan for **(A)** natural disasters and **(B)** communicable diseases.

In Taiwan, the framework for natural disaster management comprises three levels: the central government, county governments, and township governments, as shown in Figure 5A. At the central level, the Offices of Disaster Management (ODM), the Disaster Prevention and Protection Council (DPPC), the Disaster Prevention and Protection Commission, the Disaster Prevention and Protection Expert Committee, and the National Science and Technology Center (NCDR) were established under the Executive Yuan to support policy making for disaster prevention, protection, and emergency response on a national scale. At the county and township levels, DPPCs and ODMs were also established to make policies on refugee organization, disaster reports, and victim rescue on local scales.

When disasters happen, a Central Emergency Operation Center (CEOC) is established under the commanding of ministers appointed by the convener of central DPPC according to the characteristics of disaster. For a compound disaster associated with different ministries, multiple ministers can be appointed as associated commanders at the same time to provide necessary support. After the establishment of the CEOC, the county and township governments are immediately notified to establish corresponding emergency operation centers locally.

According to the CDCA, the disaster management framework for communicable diseases also comprises three levels: the central government, regional networks, and county governments, as shown in Figure 5B. The MOHW is in charge of disaster management in the central government to prevent and control communicable diseases on a national scale. Unlike with natural disasters, disaster management for communicable disease lacks the township government level but has an additional regional level lying between the central and county government levels. At the regional level, neighboring counties are grouped up into six regional medical networks to set up communicable disease isolation wards and coordinate essential medical supplies for communicable disease control. At the county government level, health bureaus are responsible for implementing the policies and plans formulated by the TCDC, and conducting disease control measures commissioned by the NHCC.

According to the CDCA, communicable diseases are classified into five categories according to the level of fatality rate, incidence rate, and transmission speed. When communicable diseases occur or are expected to occur, local governments should immediately report to the TCDC and take necessary countermeasures in accordance with their authority and responsibilities. In consideration of the severity of epidemic conditions, the NHCC may establish a Central Epidemic Command Center (CECC) to integrate resources, organizations, and personnel across different governmental levels. Regional medical networks and county governments should establish relevant epidemic command centers to execute the instructions from the CECC.

### Emergency Operation

**Figure 6:**
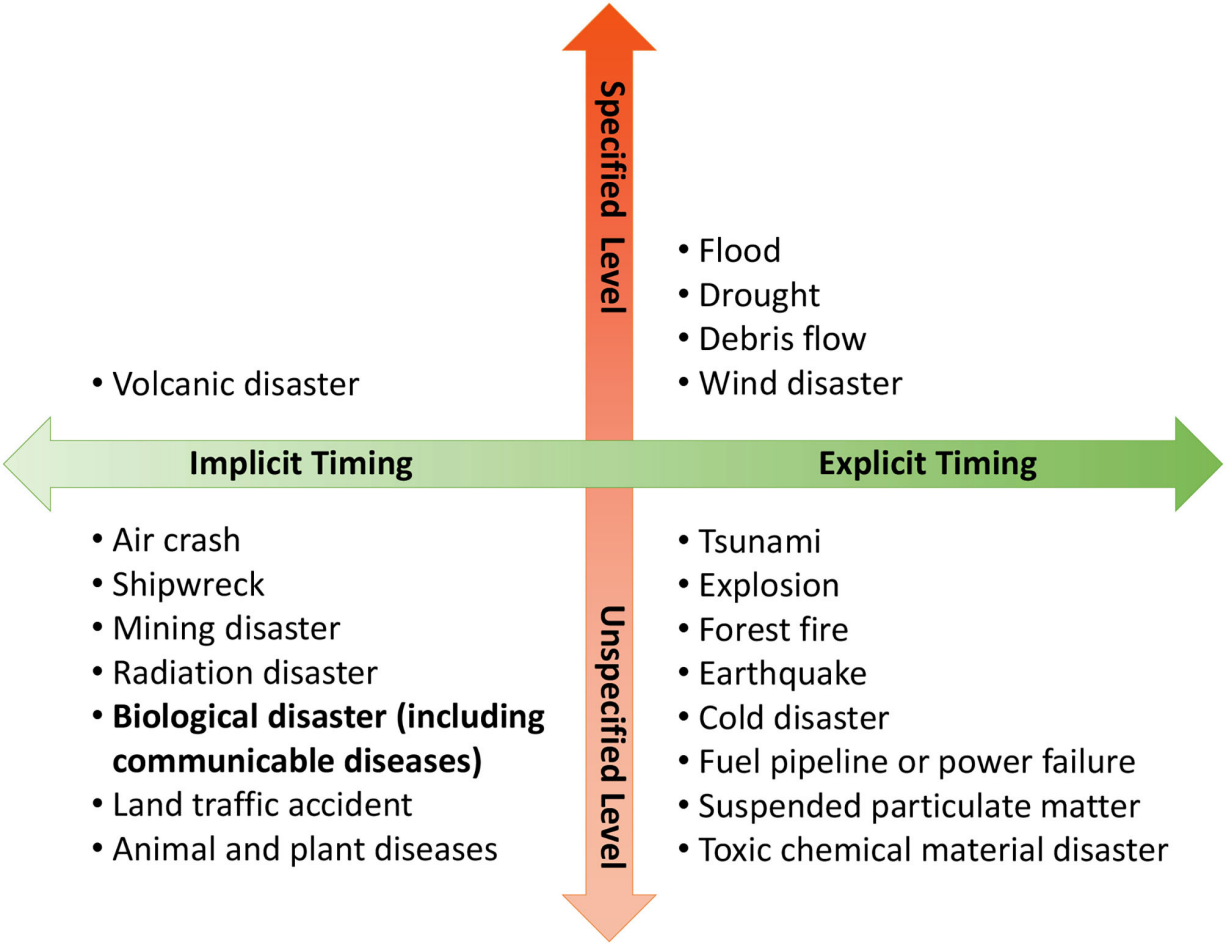
Timing and level to establish central emergency centers for different disasters.

For different kinds of disasters, the timing and levels for the establishment of CEOC are illustrated in Figure 6. Some disasters, such as floods, droughts, and debris flows, are more predictable and observable through scientific analysis, so they have explicit timing for establishing a specific level of CEOC. Some disasters, such as tsunamis and earthquakes, can be observed but happen too fast to classify emergency levels. Some disasters, such as radiation and biological disasters, are invisible and therefore unpredictable for defining the timing and levels for emergency operation.

**Figure 7:**
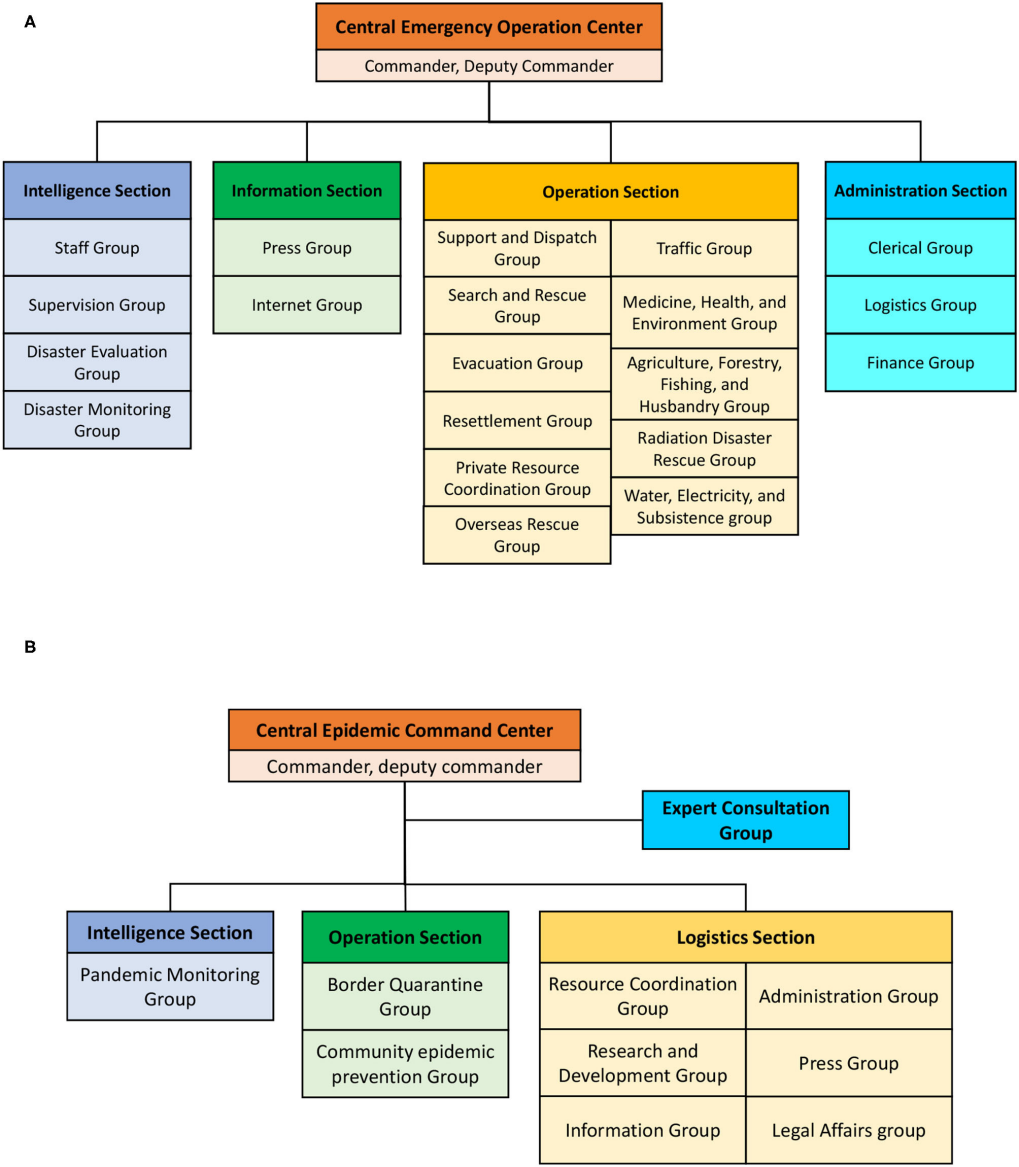
Organizations of the central emergency operation center for **(A)** natural disasters and **(B)** communicable diseases.

The organizations of the CEOC for natural disasters are displayed in Figure 7A, which comprises four sections and 20 groups. Each group comprises serval agencies with a leader to fulfill specific tasks for disaster emergency operations. For example, the disaster evaluation group, which is responsible for data gathering, damage analysis, early warning, and decision support, is led by the NCDR with members of the MOI, the MOEA, the Council of Agriculture, the Environmental Protection Administration, the Council of Indigenous People, the National Fire Agency, the Construction and Planning Agency, the Central Weather Bureau, the Directorate General of Highways, and the Ministry of Transportation and Communications. During an emergency period, the agencies from different groups station in the CEOC and work in shifts to provide all necessary support. Information and resources are constantly exchanged and updated through intergroup meetings.

Since the COVID-19 pandemic is classified as a kind of communicable disease and is governed by the MOHW, the MOHW established the CECC in January 2020 with the organizations shown in Figure 7B. Compared with the CEOC for natural disasters, the organizational structure of the CECC is more flexible and less complicated than that of the CEOC. In the intelligence section, tasks related to pandemic monitoring are directed by the TCDC in cooperation with the Department of Policy Planning and the Ministry of Foreign Affairs only. Unlike the CEOC for natural disasters, the agencies required to station in the CECC are not explicitly regulated and can change with the circumstances according to the commander's decision. This arrangement might be efficient and effective, but the information seems not be transparent enough to the stakeholders, and the performance of the CECC may rely too much on the commanders' experience.

## Lessons Learned

### Fill in the Missing Links

The enactment and enforcement of laws are especially important for creating a supportive environment for disaster management (23). As mentioned earlier, the disaster management framework for communicable diseases is regulated by the CDCA in Taiwan, which does not include the township government level as it does for natural disasters. However, in response to the COVID-19 pandemic, township officials are required to assist investigations and arrange medical treatment for people with respiratory tract symptoms during the period of home quarantine. Without being explicitly tasked in the framework or regulated by laws, township officials may lack adequate training and easily get confused when conducting the tasks. As stated by one of the interviewees from the local government, local health centers and healthcare workers are the front lines that need to be included in the overall plan, such as DPPP and CDCA, for adequate allocation of resources.

In contrast, the disaster management framework for natural disasters is insufficient at the regional scale because regional networks are not included in the DPRA. During the COVID-19 pandemic, the regional networks played a crucial role in responding to the nosocomial infections occurred in January 2021 by receiving patients from different hospitals. The benefits and coordinating functions of regional networks have been positively agreed by all the interviewees. Without regional networks, the resources across counties cannot be effectively coordinated if a disaster affects a region across counties. For example, during the cornstarch explosion disaster in the Formosa Fun Coast Waterpark in 2015, the local county did not have enough ambulances to transport victims and some requests for assistance from neighboring counties were delayed (9). This finding is supported by the United Nations Development Programme (24), ‘*the public authorities, civil servants, media, private sector, and civil society should coordinate at community, national, and regional levels in order to manage and reduce disaster and climate related risks*.' Overall, regarding both natural disasters and communicable diseases, the missing links in the disaster management systems need to be addressed.

Moreover, as pointed out earlier and shown in Figure 4, DPRA regulates biological disasters, but policy plaining and emergency response for communicable diseases should follow the instructions of the CDCA. This created confusions. For instance, the timing for establishing a local epidemic command center is described inconsistently in CDCA and DPPP — the former authorizes local governments to decide the timing when there is a need but the latter regulates that the a epidemic local command center must be established immediately once a central one is established. According to our interviewees from both the central and local governments, the establishment and operations of emergency response center and epidemic command center are apparently two entirely different systems. Although they do not see significant drawbacks in running the two systems separately, they do have different roles and tasks that are overlapped or conflicted in dealing with these two disasters.

### Passing Down the Knowledge

Moe et al. (25) indicated that disaster management practitioners should be innovative and adopt the best practices based on the experience and lessons from previous events. RICS et al. (26) emphasized that disaster recovery experience should be applied to improve the resilience of communities and to reduce disaster risks in the future. Mohanty et al. (27) indicated that knowledge can be divided into explicit and tacit knowledge. Explicit knowledge can be accessed by anyone through books, pictures, or guidelines, but tacit knowledge is learned from individual experience and can be lost with the person possessing it. In the last half century, the disaster management systems in Taiwan evolved with the knowledge learned from devastating disasters through the explicit amendment of laws to establish the ODM and NHCC after Typhoon Morakot in 2009 and the SARS pandemic in 2003, respectively.

However, as indicated in previous sections, the timing, level, and organization for establishing a CECC are not explicitly stated in the CDCA. This gave the commander more authority to adjust the operation systems according to the rapidly changing situations of the COVID-19 pandemic. However, this has pros and cons because the performance of disaster operation is highly dependent on the commander's experience and knowledge. In contrast, the operation of a CEOC for natural disasters is explicitly regulated in the DPRA, which may be less flexible, but the knowledge inherent in the regulations can be passed down to different commanders. To prepare for the next global pandemic, the experience gained from the fight against the COVID-19 pandemic must be transformed to explicit knowledge or regulations that can be accessed by others.

### Enhance Disaster Risk Governance

According to the guidelines of the Sendai framework (28), the governance of disaster risk is emphasized, which includes the understanding of hazard, vulnerability, and exposure to disasters; the recognition of stakeholders' roles; and the resilience of health infrastructure. The disaster risk management cycle proposed by RICS et al. (26) indicated that sustainable development can be achieved by reducing the risk and vulnerability of local communities in the pre-disaster phase. To achieve this, the government must establish appropriate frameworks of laws, regulations, and policies to define the roles and responsibilities of both public and private sectors. However, through the comparison between the DPRA and CDCA, we found that both fundamental laws lack the considerations of vulnerability assessment, risk communication, and recognition of stakeholders' roles. Relevant amendments should be made accordingly to enhance disaster risk governance in the future.

## Conclusions

Although Taiwan has been one of the best performers in the world in response to the COVID-19 pandemic, the disaster management systems for communicable diseases still have room for improvement through a comparison with those for natural disasters. Being regulated by different laws, the disaster management systems for communicable diseases and natural disasters are different in terms of framework, plans, and emergency operations. Compared with that for natural disasters, the framework for communicable diseases lacks the township level, which may have resulted in the undertraining of frontline staff in assisting people with respiratory tract symptoms during the COVID-19 pandemic. In contrast, the framework for communicable diseases possesses regional networks that efficiently coordinated the medical resources across counties during COVID-19 pandemic. For emergency response, the operation center for communicable diseases is more flexible by giving the commander more authority to adjust the timing, level, and organization according to the circumstances. However, this also implies that the performance of emergency operation is more dependent on the commander's level of experience. Finally, both fundamental laws for natural disasters and communicable diseases should be amended by including concepts concerning disaster risk governance, such as vulnerability assessment, risk reduction, and sustainable development.

## Data Availability Statement

The original contributions presented in the study are included in the article/supplementary material, further inquiries can be directed to the corresponding author.

## Author Contributions

W-LL, S-HY, and C-WL: analysis. H-WW and J-HJ: review and editing: H-WW and C-LS: supervision. H-WW: project administration. All authors contributed to the article and approved the submitted version.

## Funding

This research was funded by the Ministry of Science and Technology [grant number MOST 109-2327-B-006-005-].

## Conflict of Interest

The authors declare that the research was conducted in the absence of any commercial or financial relationships that could be construed as a potential conflict of interest.

## Publisher's Note

All claims expressed in this article are solely those of the authors and do not necessarily represent those of their affiliated organizations, or those of the publisher, the editors and the reviewers. Any product that may be evaluated in this article, or claim that may be made by its manufacturer, is not guaranteed or endorsed by the publisher.
